# Prospective Identification of Glioblastoma Cells Generating Dormant Tumors

**DOI:** 10.1371/journal.pone.0044395

**Published:** 2012-09-06

**Authors:** Ronit Satchi-Fainaro, Shiran Ferber, Ehud Segal, Lili Ma, Niharika Dixit, Ambreen Ijaz, Lynn Hlatky, Amir Abdollahi, Nava Almog

**Affiliations:** 1 Department of Physiology and Pharmacology, Sackler School of Medicine, Tel-Aviv University, Tel-Aviv, Israel; 2 Center of Cancer Systems Biology, Steward Research & Specialty Projects Corp., St. Elizabeth’s Medical Center, Tufts University School of Medicine, Boston, Massachusetts, United States of America; 3 Department of Radiation Oncology, German Cancer Research Center and University of Heidelberg Medical School, Heidelberg, Germany; University of Navarra, Spain

## Abstract

Although dormant tumors are highly prevalent within the human population, the underlying mechanisms are still mostly unknown. We have previously identified the consensus gene expression pattern of dormant tumors. Here, we show that this gene expression signature could be used for the isolation and identification of clones which generate dormant tumors. We established single cell-derived clones from the aggressive tumor-generating U-87 MG human glioblastoma cell line. Based only on the expression pattern of genes which were previously shown to be associated with tumor dormancy, we identified clones which generate dormant tumors. We show that very high expression levels of thrombospondin and high expression levels of angiomotin and insulin-like growth factor binding protein 5 (IGFBP5), together with low levels of endothelial specific marker (ESM) 1 and epithelial growth factor receptor (EGFR) characterize the clone which generates dormant U-87 MG derived glioblastomas. These tumors remained indolent both in subcutaneous and orthotopic intracranial sites, in spite of a high prevalence of proliferating cells. We further show that tumor cells which form U-87 MG derived dormant tumors have an impaired angiogenesis potential both *in vitro* and *in vivo* and have a slower invasion capacity. This work demonstrates that fast-growing tumors contain tumor cells that when isolated will form dormant tumors and serves as a proof-of-concept for the use of transcriptome profiles in the identification of such cells. Isolating the tumor cells that form dormant tumors will facilitate understanding of the underlying mechanisms of dormant micro-metastases, late recurrence, and changes in rate of tumor progression.

## Introduction

A dormant phase during tumor progression is highly prevalent, yet it is one of the most neglected areas in cancer research and the associated biological mechanisms are still mostly unknown [1], [2]. Cancer dormancy is a stage in which tumors are kept occult and asymptomatic for a prolonged period of time [3], [4]. It is present as one of the earliest stages in tumor development, as micro-metastasis in distant organs, and as minimal residual disease left after surgical removal or treatment of primary tumors. Dormant tumors are usually only a few millimeters diameter in size and are, therefore, undetectable by most imaging technologies currently in use [5], [6]. They can, however, switch to become fast-growing, clinically-apparent, and potentially lethal.

Since delayed disease recurrence, common in breast cancer, colon cancer and other tumor types, can be explained by the concept of tumor dormancy [7], [8], eradicating dormant tumors is currently a major challenge in cancer treatment [9]–[12]. Tumors can remain occult and asymptomatic for years, or even decades, while certain molecular and cellular mechanisms either halt, or are insufficient to enable, tumor progression and mass expansion. Clinical data and experimental models have led to the development of the concepts of cellular dormancy [13]–[16] and tumor dormancy [17]–[20]. Tumor cell dormancy is observed when solitary disseminated cancer cells either circulate in the blood system or settle at secondary sites, and is often associated with quiescence. Whereas, tumor dormancy is observed when tumors, as clusters of cells, do not expand in size over a long period of time. Clearly, dormancy of cancerous lesions depends on crucial signals from the microenvironment and the tumor stroma [4], [16], [18], [21]–[28]. Such signals can induce tumor cell quiescence. Alternatively, systemic influences – such as the immune system of the host, hormonal control, or the blockage or insufficiency of tumor angiogenesis potential – can result in dormant tumors in which cell proliferation is balanced by cell death.

A lack of suitable experimental models and limited clinical access to dormant tumors are two of the major obstacles in the advancement of research on tumor dormancy [29]. We have previously established *in vivo* models of human breast cancer, glioblastoma, osteosarcoma, and liposarcoma dormancy in severe combined immunodeficient (SCID) mice [30], [31]. These models were all derived from human tumor cell lines isolated from cancer patients and no artificial genetic modifications were made to generate the cell lines that form dormant or fast-growing tumors when injected into SCID mice. Tumor dormancy in these models was associated with an impaired angiogenic potential resulting in a delayed expansion of tumor mass. A high proliferation rate of tumor cells in dormant tumors is balanced by apoptosis and cell death. Using these models, we have shown that viable and metabolically-active, non-angiogenic, microscopic dormant tumors can reside in mice for very long periods of time until they spontaneously switch to become fast-growing, angiogenic tumors [23], [30].

Next, we sought to identify the molecular determinants of human tumor dormancy. Using genome-wide expression profiling assays to compare gene expression profiles in dormant and fast-growing tumors from our human breast cancer, glioblastoma, osteosarcoma, and liposarcoma models, we looked for genes with similar patterns of expression across all tumor types. The consensus signature of human tumor dormancy was then determined based on genes that were differentially expressed between dormant and fast-growing tumors in the same pattern in all tumor types analyzed [23]. For example: in all dormant tumors, high expression of thrombospondin and angiomotin with concomitant low expression of CD73 and epidermal growth factor receptor (EGFR) were observed.

Tumor cells are well known to be heterogeneous with respect to a wide variety of characteristics such as metastatic activity, angiogenic potential, proliferation rate, and enzymatic activity [19]. Here, we set out to test whether the tumor dormancy gene signature that we previously identified can be used for isolation of tumor cells that will form non-angiogenic dormant tumors. Hence, this approach can lead to further and deeper understanding of the molecular mechanisms underlying human tumor dormancy.

## Results

**Figure 1:**
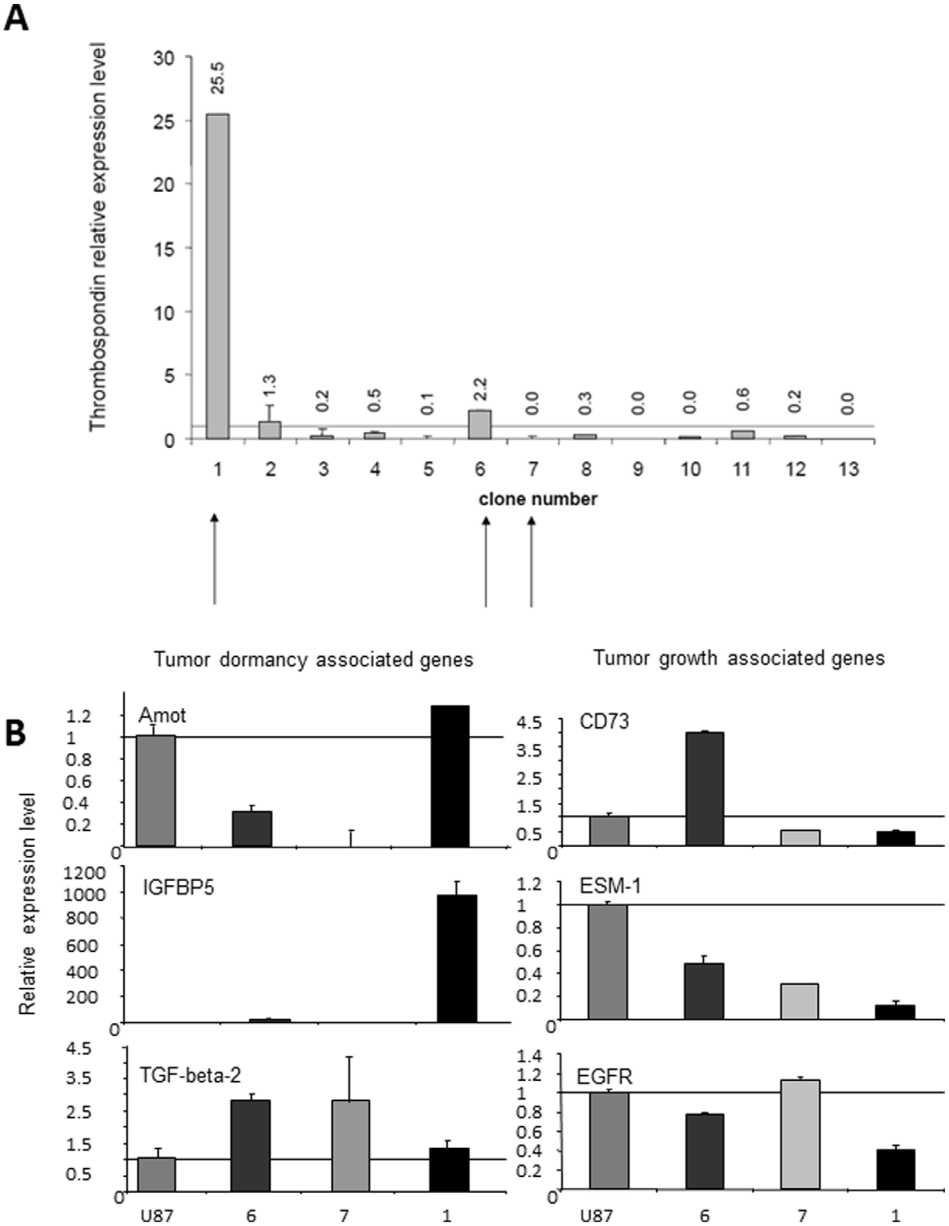
Gene expression analysis of single cell-derived clones from U-87 MG glioblastoma cell line. All RT-PCR measurements were normalized according to expression in the parental U-87 MG cell line. **A.** Thrombospondin-1 (TSP-1) relative level in U-87 MG derived clones. **B.** Expression level of genes previously shown to be upregulated in dormant tumors is shown on the left panel. Expression level of genes previously shown to be upregulated in fast-growing tumors is shown on the right panel.

Single cell derived clones were generated using a limiting dilution method from the parental U-87 MG human glioblastoma cell line. Thirteen clones were chosen according to similar rapid kinetics of colony formation in tissue culture wells. RNA was extracted from each clone and the relative expression level of Thrombospondin (TSP-1), a well-known endogenous inhibitor of angiogenesis that has been shown to be elevated in all dormant tumors [23], was determined using real time PCR (Fig. 1A). When compared with the expression level of the parental U-87 MG cell line, most (10 out of 13) of the clones had lower TSP-1 expression, while only 3 clones (#1, #2 and #6) had elevated TSP levels. Clone #1 had a significant increase in TSP level (over 25-fold higher expression than in parental U-87 MG cell line).

Since a high TSP level could suggest slow kinetics of tumor growth, we chose to focus our analysis on three clones with varying TSP levels: Clone #1, with the highest TSP level, Clone #6 with an intermediate TSP level, and Clone #7 with a very low TSP level (marked by arrows in Fig. 1A). We postulated that Clone #1 might generate dormant tumors, whereas clones #6 and #7 would generate intermediate or fast-growing tumors, respectively. We then continued to analyze the expression levels of several tumor dormancy-associated genes we had previously identified [23]. First, we tested the expression levels in the three clones of the additional genes that were previously shown to be upregulated in dormant tumors (Fig. 1B). Clearly, angiomotin (Amot) and IGFBP5 levels were upregulated only in Clone #1. Notably, IGFBP5 expression was around 1000-fold higher than in the parental U-87 MG cell line. Expression of TGF-β2 was upregulated in all clones tested.

Genes previously shown to be elevated in fast-growing tumors were expected to be observed as downregulated in tumor cells that form dormant or slow-growing tumors. Such downregulation was indeed observed in Clone #1 for CD73, EGFR, and most significantly for ESM-1 (Fig. 1B), strengthening our prediction that this clone could generate dormant tumors.

**Figure 2:**
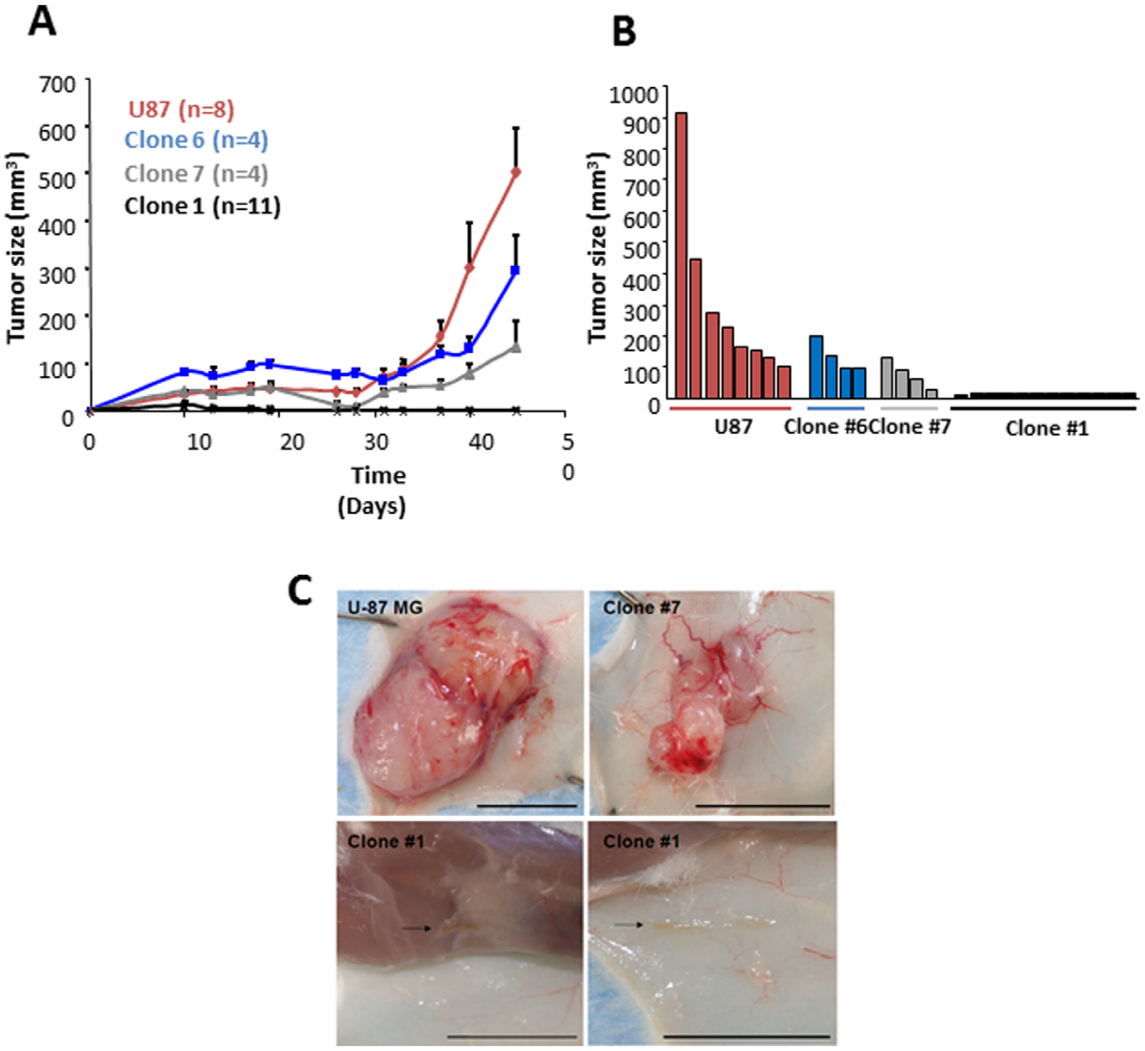
Comparison of tumor growth patterns and characteristics. **A.** Tumor growth kinetics of U-87 MG derived clones in SCID mice. **B.** Tumor size of U-87 MG clones 40 days following tumor cell inoculation. While tumors generated from U-87 MG parental cell line, Clone #6, or Clone #7 were clearly detected, the tumors generated from Clone #1 were undetectable by gross examination. Tumor growth analysis comparing U-87 MG and Clone #1 derived tumors was repeated in independent settings three times. **C.** Tumors generated from the U-87 MG parental cell line and derived clones 47 days after injection. Scale bars represent 1 cm.

Tumor growth patterns were then analyzed in SCID mice. Equal numbers of cells were injected subcutaneously (s.c.) from each clone and from the parental U-87 MG cell line, and tumor growth was monitored (Fig. 2A). As expected, the parental U-87 MG cells generated very small tumors (volume below 100 mm^3^), which after 3–4 weeks initiated rapid growth. Similar ‘bi-phasic’ growth kinetics were observed for tumors generated from clones #6 and #7. Although Clone #6, which had an intermediate level of TSP, initially formed tumors larger than the parental cell line, its tumors grew slower in the rapid growth phase. Clone #7, which had a very low level of TSP, formed tumors smaller than those generated by the parental cell line or by Clone #6. Importantly, Clone #1 formed dormant tumors which remained indolent and were barely detectable by gross examination throughout the experiment (Fig. 2B). This confirmed our hypothesis that the parental U-87 MG cell line contains cells which when isolated will form dormant tumors, and that Clone #1 was generated from such cells.

At the end point of the experiment, tumors generated by clones #6 and #7 were clearly smaller than those generated by the parental U-87 MG cells. Although smaller in mass, Clone #6 and Clone #7 tumors were highly vascularized and tightly capsulated, similar to tumors generated from parental U-87 MG cells (Fig. 2C). In contrast, tumors generated from Clone #1 could be detected only after flipping the skin and seemed avascular. These tumors were occasionally found attached to the muscle tissue instead of the skin, like most tumors from U-87 MG, Clone #6, and Clone #7 (Fig. 2C).

The fate of indolent tumors generated by Clone #1 was analyzed by following their tumor growth over a prolonged period of time lasting more than 200 days (Fig. S1). As expected, while U-87 MG tumors grew rapidly in the first 3–4 weeks after inoculation, tumors from Clone #1 remained undetectable for over 70 days. Three of the four tumors from Clone #1 eventually emerged from dormancy and initiated growth at 81, 122, and 127 days post inoculation (Fig. S1). One mouse injected with Clone #1 cells never developed any detectable tumors during the 270 days of the experiment (data not shown). Tumors that originated from Clone #1 cells remained at the site of injection in a constant small size without expanding in mass for a long period of time (i.e., dormant). Importantly, once these tumors emerged from dormancy and started growing, the growth rate could be as rapid as in the parental U-87 MG cell line derived tumors.

**Figure 3:**
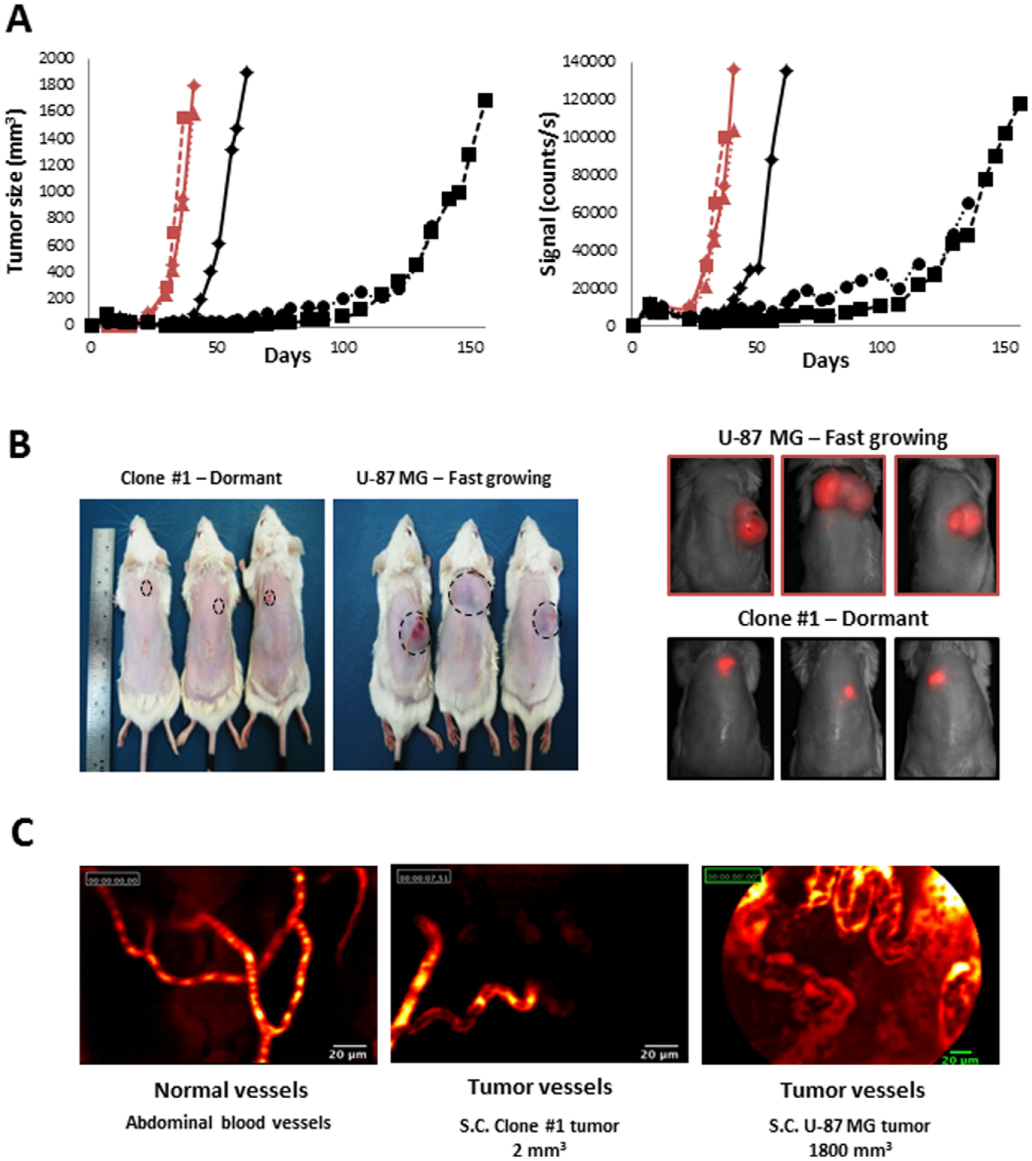
Tumor growth patterns of mCherry-labeled tumor cells. **A.** Size of U-87 MG (red, n = 3) and Clone #1 (black, n = 3) tumors measured by caliper (**left panel**) and by non-invasive CRI Maestro™ imaging system (**right panel**). **B.** Forty days post subcutaneous inoculation of mCherry-labeled U-87 MG and Clone #1 cells into SCID mice, U-87 MG cells established vascularized and palpable tumors (∼1200 mm^3^), whereas Clone #1 tumors remained avascular and non-palpable (**left panel**), but detectable by non-invasive CRI Maestro™ imaging system (**right panel**). **C.** Fiber confocal microscopy imaging of U-87 MG and Clone #1 tumor vasculature, as well as abdominal blood vessels, on day 40.

To evaluate tumor properties in their orthotopic microenvironment in a non-invasive manner, both Clone #1 and U-87 MG parental cells were infected with mCherry as previously described [32]. Then, in order to assure that the infection did not alter tumor characteristics, SCID mice were inoculated s.c. with either cell line, and dormancy periods were monitored and compared. Tumors generated by cells from Clone #1 remained dormant and avascular for more than 70 days, while tumors generated from the parental U-87 MG cell line were highly vascularized and palpable 20 days following inoculation (Fig. 3A–3B). Following the escape from dormancy, mCherry-labeled Clone #1 tumors showed a similar tumor growth rate pattern to mCherry-labeled U-87 MG tumors (Fig. 3A–3B).

Cellvizio® imaging of the vasculature of U-87 MG tumors revealed enlarged, highly tangled, and non-continuous vessels with wider lumen and blunt ends, leakage, and sluggish blood flow. These are typical signs of the enhanced permeability and retention (EPR) effect phenomenon for macromolecules such as Dextran-FITC at 70 kDa size used here (Fig. 3C). In contrast, blood vessels in Clone #1 tumors at a size of 2 mm^3^ are almost non-appearing. For comparison, normal vessels in healthy tissues adjacent to these tumors are shown to have continuous blood flow and a regular shape with anastomosis.

**Figure 4:**
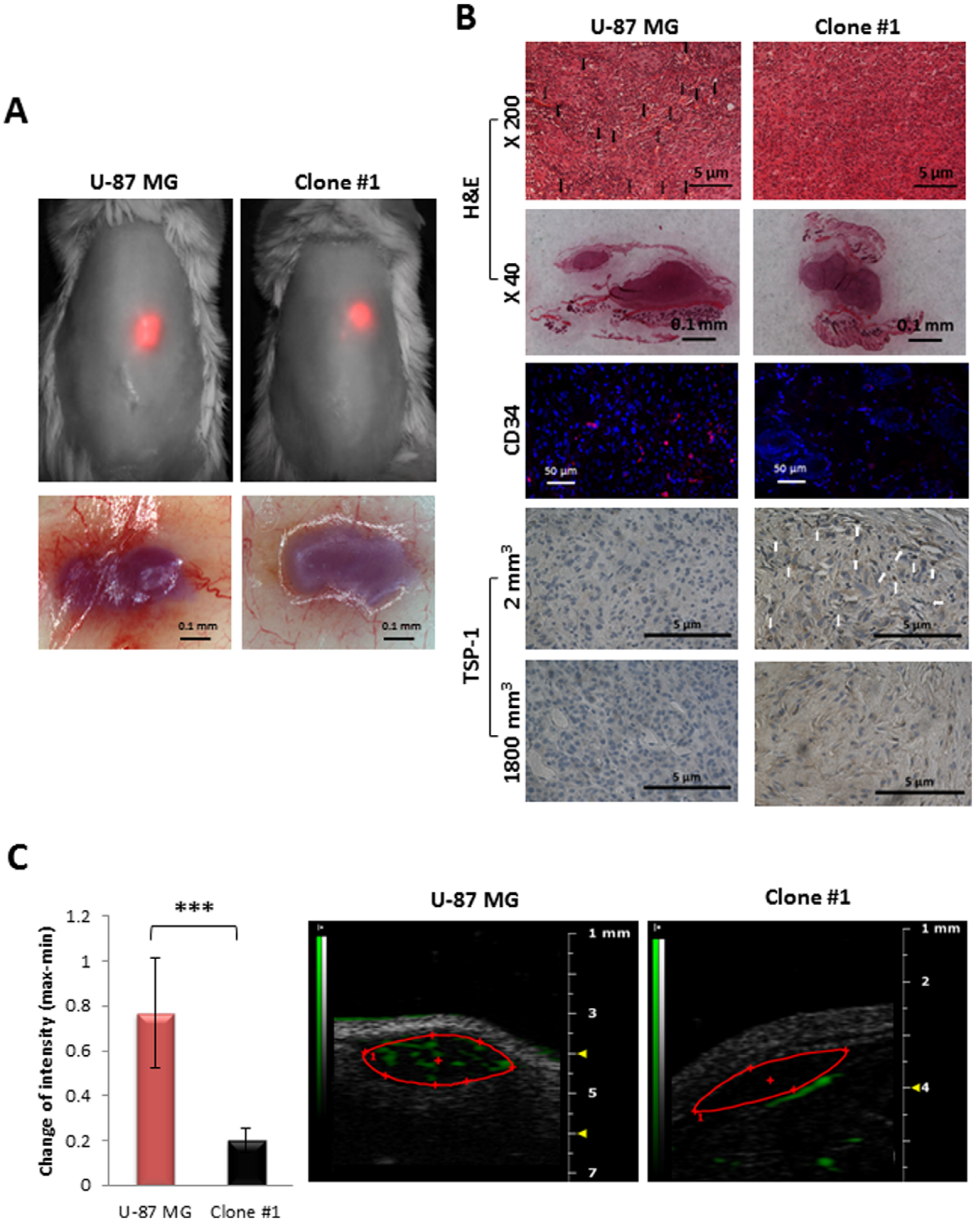
Comparison of size-matched tumors generated from U-87 MG and Clone #1 cells. **A.** Sixteen days post subcutaneous inoculation of mCherry labeled U-87 MG and Clone #1 cells, both tumor types were approximately 2 mm^3^ in diameter and detectable by non-invasive CRI Maestro™ imaging system (**upper panel**). Flipped skin of tumor-bearing mice revealed highly vascularized U-87 MG-generated tumors, while blood vessels were only detectable in the surrounding skin of Clone #1-generated tumors (**lower panel**). **B.** H&E, CD34 (merged image. The separate images are provided as Fig. S2) and TSP-1 staining of U-87 MG and Clone #1 tumor sections. TSP-1 staining was done on size-matched tumors from day 16 (2 mm^3^) and on large tumors (U-87 MG tumors at end point of experiment and Clone #1 tumors after escape from dormancy) (1800 mm^3^). **C.** Contrast-enhanced US imaging of U-87 MG and Clone #1 subcutaneous tumors show high vascularization of the U-87 MG fast-growing tumor (red bar, n = 5) compared with Clone #1 dormant tumors (black bar, n = 3) (*p* = 0.008). Data represent mean ± s.d. *** p<0.01.

To compare the angiogenic potential, *in vivo* analysis of size-matched tumors (∼2 mm^3^) from U-87 MG and Clone #1 was performed (Fig. 4). The presence of the tumors at the site of injection was validated by the mCherry fluorescent signal and later by H&E staining (Fig. 4A–4B). U-87 MG tumors were significantly more vascularized compared with size-matched tumors generated from Clone #1 cells, as observed by gross examination of the tumors after flipping the skin, by analysis of CD34 positively-stained cells and by presence of blood vessels in H&E staining (marked with arrows) in the U-87 tumor sections (Fig. 4A–4B and Fig. S2). Microbubbles contrast-enhanced ultrasound (US) imaging not only supported these results, but also emphasized the functionality of the blood vessels by extensive blood flow within U-87 MG tumors, as opposed to no detectable blood flow within Clone #1 tumors (Fig. 4C). These results are in accordance with high TSP-1 expression in tumors generated by Clone #1 compared to none in U-87 MG tumors (Fig. 4B). Interestingly, Clone #1 tumors express TSP-1 exclusively prior to the escape from dormancy, when tumor size is approximately 2 mm^3^ (Fig. 4B). Tumors generated from Clone #1 that had emerged from dormancy and reached a diameter of over 1500 mm^3^ do not express TSP-1. U-87 MG tumors do not express TSP-1 in any stage of tumor progression.

**Figure 5:**
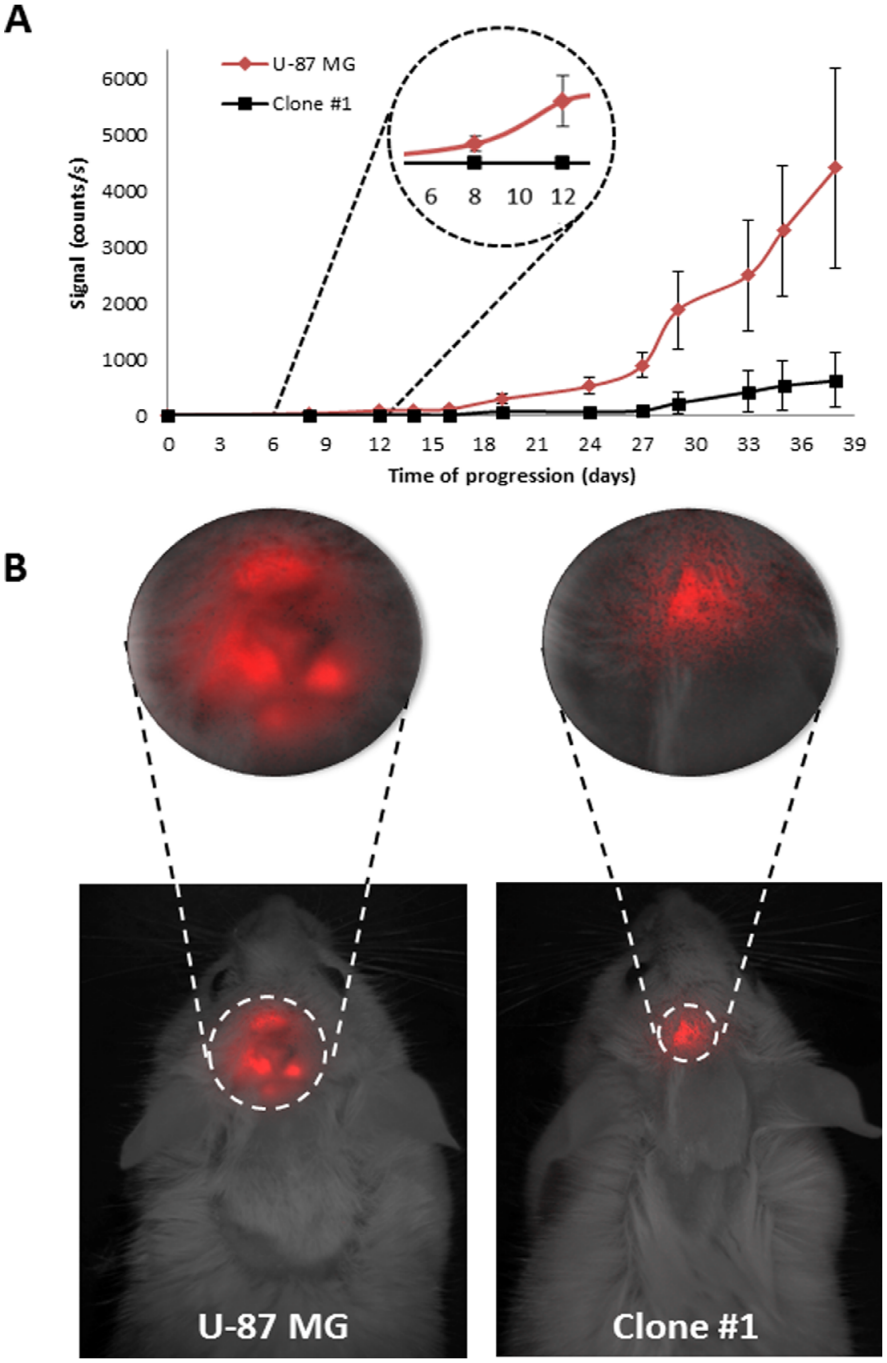
Orthotopic tumor growth patterns. **A.** Dormancy periods of mCherry-labeled U-87 MG (red, n = 6) and Clone #1 (black, n = 7) orthotopic tumors (total signal (counts/s) as measured by CRI Maestro™ imaging system). **B.** Twenty-four days post intracranial inoculation, U-87 MG tumors are significantly larger as detected by increased fluorescent signal (∼750 counts/s) compared with Clone #1 tumors (∼300 counts/s) (*p* = 0.006).

With the purpose of investigating the growth rate of orthotopic tumors, mCherry-labeled U-87 MG and Clone #1 cells were inoculated by stereotactic injections to the striatum of SCID mice. Tumor growth patterns were monitored and compared (Fig. 5A–5B). Whereas tumors originating from Clone #1 cells remained at a small size for more than 30 days, tumors originating from the parental U-87 MG cells initiated growth and mass expansion 8 days post inoculation. The dormant phenotype of tumors generated by Clone #1 does not depend, therefore, on the site of injection, but is rather an intrinsic property of the tumor cells.

**Figure 6:**
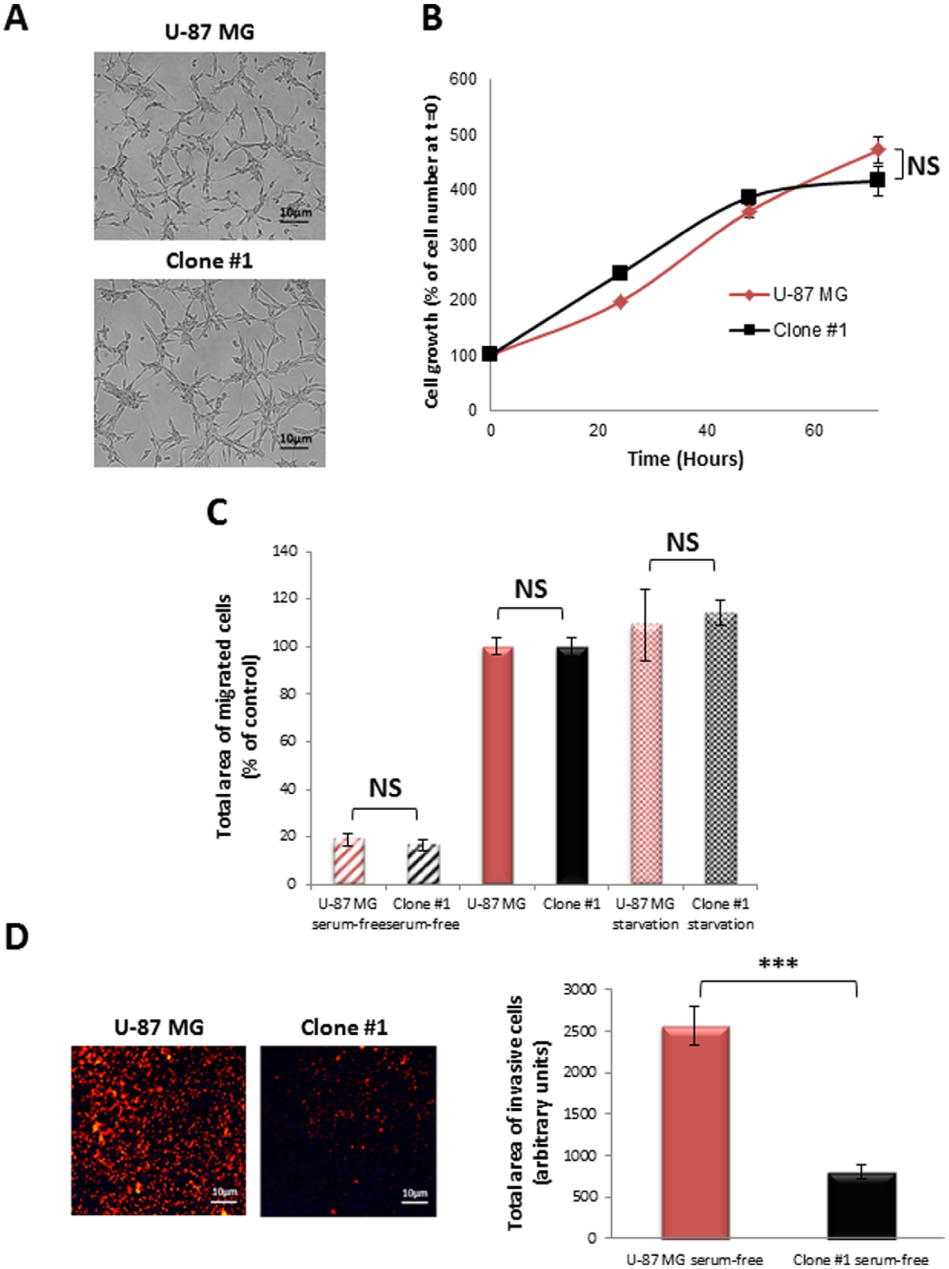
*In vitro* cellular characteristics of U-87 MG and Clone #1 cells. **A**. Representative images of cells in culture showing similar morphology. **B.** *In vitro* proliferation assay: Clone #1 (black line) and U-87 MG (red line) human glioblastoma cell lines grow at a similar rate *in vitro.* **C.** Clone #1 (black bars) and U-87 MG (red bars) cells migrate similarly toward serum-free media (striped bars), towards serum-containing media (solid bars) and following 12 h serum starvation (serum-free) conditions (dotted bars). **D.** Transendothelial migration**:** U-87 MG cells (red bar) migrate at a significantly higher rate (*p* = 1.9×10^−11^) compared with Clone #1 cells (black bar) towards serum-free media (right panel describes the quantification of representative images on the left panel). Data represent mean± s.d. from three independent experiments. NS = non-significant, *** p<0.01.

We next compared the *in vitro* proliferation kinetics, migration, invasiveness and cell morphology of U-87 MG and Clone #1 cells. No significant changes were observed in cell structure and morphology in culture (Fig. 6A). Cell growth kinetics for 72 hours were similar when U-87 MG cells were compared with Clone #1 cells (Fig. 6B). Furthermore, no difference was found between the migration ability of U-87 MG and Clone #1 cells (Fig. 6C). However, the ability of the cancer cells to migrate and invade through the endothelial cell monolayer differs significantly. The migration of U-87 MG cells through endothelial cells towards serum-free media was significantly higher compared with that of Clone #1 cells (Fig. 6D). These observations suggest that the dormancy seen in tumors generated from Clone #1 cells does not result from slower cell proliferation or migration, but could relate to their relative lack in invasion capacity as demonstrated in the transendothelial assay.

**Figure 7:**
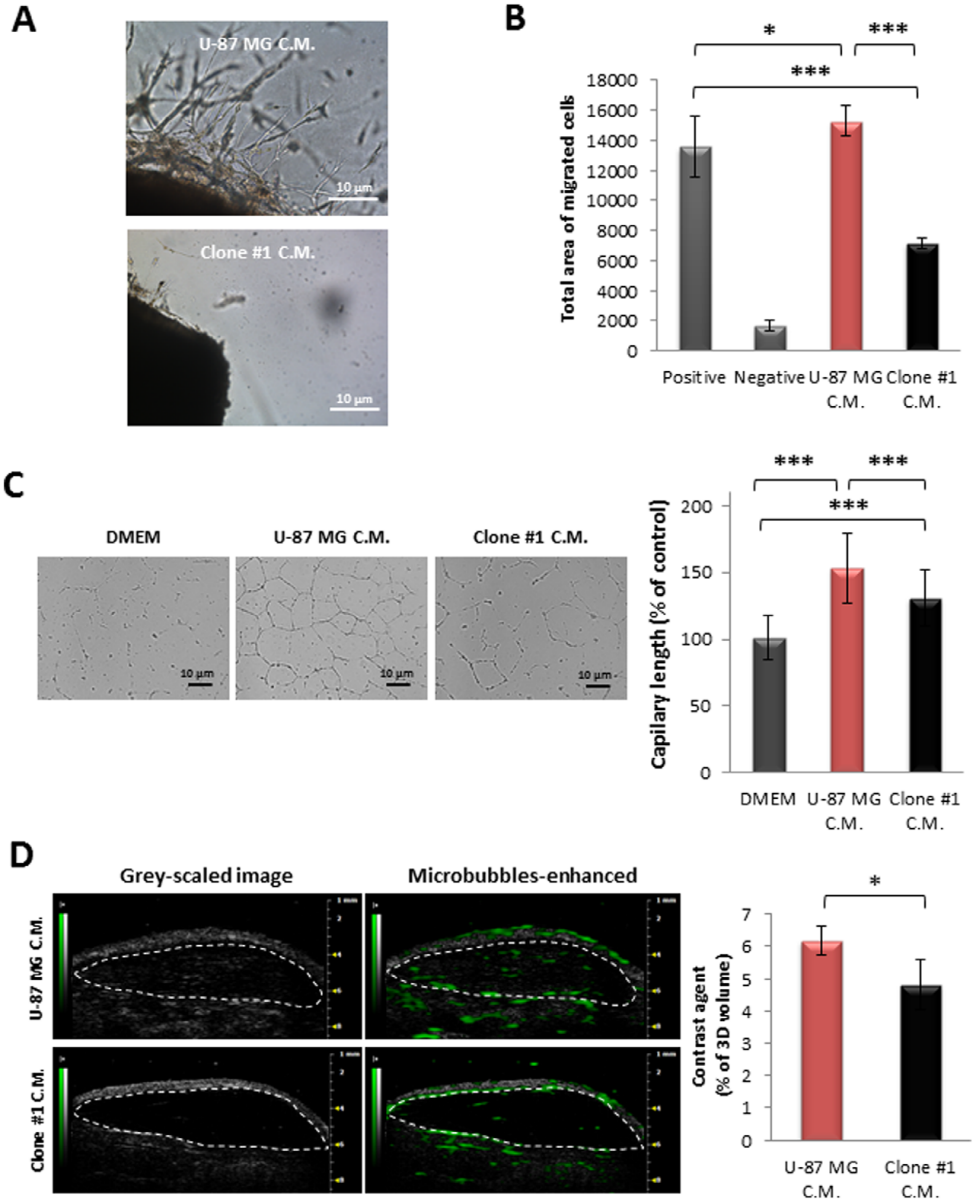
Angiogenic potential comparison between cells of U-87 MG and Clone #1. **A.** C.M. of U-87 MG cells induced extensive sprouting of endothelial cells (upper panel) from aortic rings resected from mice as compared with C.M. of Clone #1 (lower panel). **B.** HUVEC migrate towards C.M. of U-87 MG cells (red bar) at a significantly increased rate (*p* = 1.6×10^−13^) compared with that of Clone #1 (black bar). DMEM containing 10% FBS served as positive control; DMEM served as negative control. **C.** HUVEC’s ability to form capillary-like tube structures on Matrigel® is significantly higher (*p* = 0.001) in the presence of C.M. of U-87 MG cells (red bar) compared with that in the presence of C.M. of Clone #1 cells (black bar). **D.** Contrast-enhanced ultrasound imaging of subcutaneously-inoculated plugs containing C.M. media from U-87 MG cells (red bar, n = 3) showed increased vascularization compared with C.M. from Clone #1 cells (black bar, n = 3) (*p* = 0.04). Data represent mean± s.d. from three independent experiments. * p<0.05, *** p<0.01.

The angiogenic potential of the tumor cells was evaluated by comparing the effect of conditioned media (C.M.) collected from U-87 MG and Clone #1 cells on endothelial cells in a series of assays which represent different steps in the angiogenesis process (Fig. 7). Differences in the angiogenic potential of the two cell lines were demonstrated by extensive proliferation and sprouting following 7 days of incubation in the presence of C.M. from U-87 MG cells, compared with negligible sprouting in the presence of C.M. from Clone #1 cells (Fig. 7A). Also, HUVEC exhibited significantly higher migration rates towards C.M. from U-87 MG cells, compared with that towards C.M. from Clone #1 cells (Fig. 7B). Interestingly, HUVEC migrated toward C.M. from U-87 MG at a significantly faster rate than toward medium supplemented with 10% serum (positive control) (Fig. 7B). Similarly, increased numbers of tube-like structures and vascular network areas were observed in the presence of U-87 MG C.M. compared with the number found in HUVEC in the presence of both C.M. from Clone #1 cells or medium supplemented with 10% serum (Fig. 7C).

HUVEC’s ability to form capillary-like structures in the presence of C.M. from Clone #1 cells was enhanced compared with that in the presence of medium supplemented with 10% serum, indicating the presence of factors that positively affect HUVEC’s ability to form tube-like structures (Fig. 7C). The functionality of the vasculature recruited by factors secreted by the cancer cells was assessed using 3D contrast-enhanced ultra-sound analysis of s.c. Matrigel® plugs containing C.M. from the two cell lines (Fig. 7D). The vascularization within plugs containing C.M. from U-87 MG cells was significantly higher, when compared to vascularization within plugs containing C.M. from Clone #1 cells. Clearly, the level of pro-angiogenic factors that positively affect endothelial cells’ ability to proliferate, sprout, migrate, form tube-like structures and finally form functional blood vessels is higher within C.M. from U-87 MG cells as compared to C.M. from Clone #1 cells.

**Figure 8:**
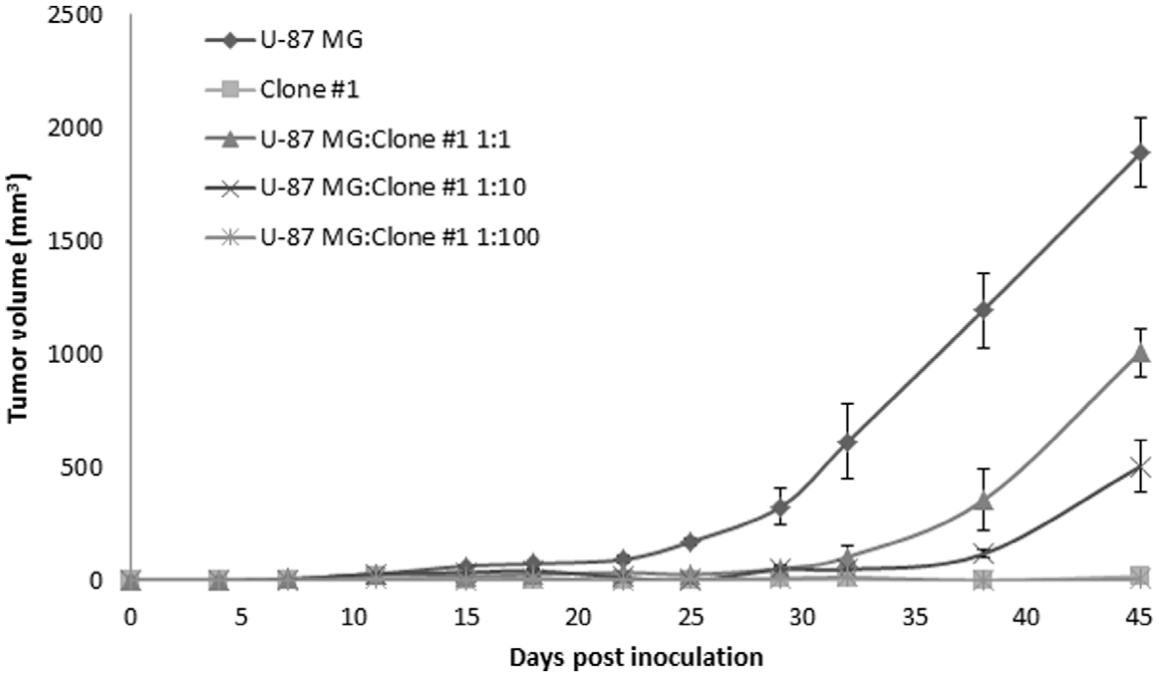
Tumor growth patterns of mixed cancer cell populations. Growth of tumors generated by only U-87 MG or Clone #1 cells was compared to that generated by mixing U-87 MG and Clone #1 cell lines in ratio of 1∶1, 1∶10, and 1∶100 (U-87 MG: Clone #1). Tumor size (3–5 mice per group) was measured by caliper.

These observations suggest that U-87 MG cells are more angiogenic than Clone #1 cells. Since the angiogenic potential of cells depends on the ratio of pro- and anti-angiogenic factors, it is possible that Clone #1 overexpresses factors which negatively affect angiogenesis. In order to assess the balance between pro- and anti-angiogenic factors (either directly or indirectly affecting endothelial cells) produced by the cancer cells, an equal total number of cells (1×10^6^) were injected s.c. into SCID mice as mixed populations of U-87 MG and Clone #1 in different ratios: 1∶1, 1∶10, and 1∶100, respectively. Tumor progression was followed and compared to the single population (Fig. 8). When 50% and 90% of cells implanted were of Clone #1, delayed escape from dormancy was observed by approximately 10 and 20 days, respectively. When the cell population consisted of 99% Clone #1 and only 1% U-87 MG cells, a dormancy period identical to that of the Clone #1 single-population was observed. This suggests the presence of anti-angiogenic factors, overexpressed mainly by Clone #1 cells.

## Discussion

We have shown that the tumor dormancy consensus gene expression signature, which we had previously identified, can be used to isolate tumor cells that have the potential to form dormant tumors. In this manuscript, we present the identification of a clone, derived from the aggressive tumor-forming U-87 MG human glioblastoma cell line, that generates dormant tumors. This supports previous indications that tumor cell populations are heterogeneous and can contain cells that, although fully transformed, have low tumorigenic potential [19].

The gene signature of dormant tumors was derived from a series of *in vivo* models of human tumor dormancy, in which dormancy was associated with impaired tumor angiogenesis. In these models, inhibition of tumor growth was linked to low microvessel density and abnormal tumor vasculature structure [23]. We decided to focus on TSP, a well-known inhibitor of angiogenesis, as the primary indicator for slow tumor growth. High levels of TSP expression, as observed in Clone #1, strongly suggested that this clone could generate dormant tumors. Kaur *et al.* [33] also demonstrated that TSP expression levels affected U-87 MG tumor growth. They showed that expression of Vasculostatin, a fragment of brain angiogenesis inhibitor-1 (BAI1) which contains five thrombospondin type 1 repeats (TSR), in U-87 MG clones strongly suppressed tumor growth and reduced the microvascular density in both s.c. and orthotopic models [33]. Further support for the possible dormant phenotype of tumors generated by Clone #1 were the concomitant overexpression of genes such as angiomotin, IGFBP5, and TGF-β2 – which were previously shown to be associated with tumor dormancy – together with down-regulation of CD73, ESM-1, and EGFR – genes associated with rapid tumor growth.

Although Clone #7 had a relatively lower level of TSP-1 compared with the parental U-87 MG cell line, it still generated tumors that grew more slowly than those tumors that were formed from the parental cell line. This could potentially result from the low expression levels of ESM-1 and CD73. Clone #6, which had moderately high levels of TSP-1, had low levels of angiomotin and IGFBP5.

Although changes in the expression levels of the genes described above were shown to be associated with tumor dormancy or growth, their exact role in regulation of dormancy is still unknown. Moreover, our initial tumor dormancy signature consisted of over 300 genes [23]. Other genes identified in the signature, could play a major role in tumor dormancy regulation and could be used for identification of additional clones. It should also be mentioned that a few tumor dormancy-associated genes that were analyzed in Clone #1 (i.e., EphA5 and IGFR1), did not show the expression pattern expected (data not shown). While compensatory and adaptive mechanisms could account for the changes in gene expression profiles observed, it is clear that the identification of clones that will form dormant tumors should rely on a group of genes. The exact combination of genes is still to be determined.

The observation that dormancy of tumors generated from Clone #1 cells is associated with impaired angiogenic potential of the tumor cells, is consistent with previous observations in other tumor dormancy models [19], [20], [23], [26], [30], [31]. Data presented in this manuscript suggest the presence of factors positively affecting the establishment of tumor vasculature (i.e., pro-angiogenic factors) within the U-87 MG tumor microenvironment, or the presence of factors negatively affecting tumor vasculature (i.e., anti-angiogenic effects) within the microenvironment of tumors generated from Clone #1 cells. The mixed population *in vivo* experiments (Fig. 8) demonstrate that the dormancy period depends on the relative ratio of Clone #1 cells. Indeed, in the scenario in which Clone #1 overexpresses anti-angiogenic factors, rather than the overexpression of pro-angiogenic factors by U-87 MG, a higher proportion of Clone #1 cells within the tumor cell population will overcome the U-87 MG fast-growing angiogenic phenotype. The fact that inhibition of tumor progression correlated with the relative proportion of cells from Clone #1 support our prediction that Clone #1 cells secrete an inhibitor of angiogenesis.

Although dormant occult tumors are highly prevalent, the cellular and molecular mechanisms underlying tumor dormancy remain to be elucidated. The molecular identification of the tumor cells that generate dormant tumors will significantly support further understanding of the regulatory network underlying tumor dormancy. This could provide an attractive venue for rational design of novel targeted therapy combinations aimed at preventing the transition of dormant tumors into fast-growing ones, and perhaps at reversing the process.

## Materials and Methods

### Ethical Statement

All animal procedures were approved and performed in compliance with the standards of Tel Aviv University, Sackler School of Medicine Institutional Animal Care and Use Committee (IACUC) and of Tufts University, St. Elizabeth’s Medical Center IACUC.

### Materials

Dulbecco’s modified Eagle’s medium (DMEM), Fetal Bovine Serum (FBS), Penicillin, Streptomycin, Nystatin, and L-glutamine were purchased from Biological Industries (Kibbutz Beit Haemek, Israel). Matrigel®, growth factor-reduced Matrigel and Bacto™ Agar were from BD Bioscience (Franklin Lake, NJ, USA). Boyden chambers 8 µm were from Transwell-Costar Corp. Hema 3 Stain System was from Fisher Diagnostics. EGM-2 medium was from Cambrex, USA and endothelial cells growth supplement (ECGS) from Zotal, Israel.

### Cell Lines, Tissue Culture and Surgical Specimens

Human glioblastoma (U-87 MG) cells and human embryonic kidney 293T (HEK 293T) cells were obtained from the American Type Culture Collection (ATCC, Manassas, VA, USA). Human umbilical vein endothelial cells (HUVEC) were purchased from Lonza, Switzerland. Dormant and angiogenic fast-growing populations were generated and maintained as previously described [23], [30]. U-87 MG and 293T cancer cells were grown in DMEM supplemented with 10% FBS, 100 mg/mL Penicillin, 100 U/mL Streptomycin, 12.5 U/mL Nystatin, and 2 mM L-glutamine. HUVEC were cultured in EGM-2 medium (Lonza, Switzerland). All Cells were grown at 37°C; 5% CO_2_.

### Generation of mCherry-labeled U-87 MG Human Glioblastoma Cell Lines

mCherry-labeled U-87 MG human glioblastoma cell lines were generated as previously described [32]. Briefly, human embryonic kidney 293T (HEK 293T) cells were co-transfected with pQC-mCherry and the compatible packaging plasmids, pMD.G.VSVG and pGag.pol.gpt. Forty-eight hours following transfection, the pQC-mCherry retroviral particles-containing supernatants were collected and filtered (0.45 µm). U-87 MG and Clone #1 human glioblastoma cells were infected with the retroviral particles-containing media and mCherry positive cells were selected 48 hours following the infection by puromycin (3 µg/ml) resistance.

### Total-RNA Extraction and RT-PCR

Total-RNA, including miRNAs, was isolated using TRIzol (Invitrogen) according to the manufacturer’s protocol. RNA integrity and concentration was determined using RNA 6000 Nano Lab on Chip kits and Agilent 2100 Bioanalyzer (Agilent, CA, USA). RNA (2 µg) was subjected to reverse transcription reaction (RT). RT reactions were carried out using the high capacity cDNA reverse transcription kit (Applied Biosystems) according to manufacturer’s protocol. Real-time PCR was done using TaqMan Gene Expression Assays (Applied Biosystems) kits. Primers were used according to our prior publication [23]. PCR amplifications were performed as multiplex, together with 18 s probe as an internal control. For low copy number genes, single PCRs were performed. Results were then normalized according to the internal control of B2M VIC/MGB probe (Applied Biosystems).

### Cell Proliferation Assay

U-87 MG cells were plated into 24-well plates (15×10^3^ cells/well). Following incubation (37°C; 5% CO_2_), cells were counted by Coulter Counter (Beckman Coulter) every 24 hours during four days.

### Cancer Cells Migration Assay

Cell migration assay was performed using transwells chambers with pore size of 8 µm (Costar Corp., Corning, NY, USA). U-87 MG and Clone #1 were starved in 2% FBS containing growth media for 12 hours. U-87 MG and Clone #1 cells (1×10^5^ cells), starved and non-starved, were added to the upper chamber of 10 µg/ml fibronectin-coated 24-well transwells chambers. Following 2 hours of incubation, 10% FBS containing media or serum-free media were added to the lower chamber.

Cells were allowed to migrate to the lower chamber for an additional 4 hours, followed by fixation using methanol and staining (Hema 3 stain system). The stained migrated cells were imaged using a Nikon TE2000E inverted microscope integrated with Nikon DS5 cooled CCD camera by 10 × objective, brightfield illumination. Migrated cells from the captured images per membrane were counted using NIH imageJ software. Migration was normalized to percent migration, with 100% representing migration towards FBS.

### Transendothelial Migration Assay

Transendothelial migration assay was performed using transwells chambers with pore size of 8 µm (Costar Corp., Corning, NY, USA). HUVEC (25×10^4^ cells) were grown confluently in the upper chamber of inserts pre-coated with 10 µg/ml fibronectin inserted into 24-well transwells. Following 24 hours incubation, HUVEC monolayer was washed and 5×10^4^ mCherry-labeled U-87 MG or Clone #1 cells were added to the upper chamber. After 1 hour of incubation, serum free media was added to lower chamber. Cells were allowed to migrate to the lower compartment for an additional 6 hours and then fixed using 3.7% formaldehyde. The fluorescence of the migrated cells were imaged using Nikon TE2000E inverted microscope integrated with Nikon DS5 cooled CCD camera by 10 × objective. Fluorescence signal was measured using NIH ImageJ software.

### Conditioned Media Preparation

Conditioned media for HUVEC migration and capillary-like tube formation assays was collected from confluent 12 cm^2^ tissue culture plates of either U-87 MG or Clone #1 following 48 hours incubation at 37°C in 5% CO_2_. Prior to the experiments, the conditioned media was filtered through 0.45 µm syringe filter to remove cells and debris.

### Aortic Ring Assay

Aorta was resected from a Balb/c mouse, sliced to 1 mm pieces and placed in a 48-well plate coated with Matrigel® basement membrane (150 µl/well; 10 mg/ml) on ice following 30 min incubation at 37°C. Additional Matrigel® basement membrane (100 µl/well; 10 mg/ml) was added and allowed to polymerize at 37°C for 30 min. Conditioned media from either U-87 MG or U-87 MG-Clone #1 (300 µl) was added. Sprouting of endothelial cells from the resected aorta slices was imaged following 7 days incubation in 37°C using Nikon TE2000E inverted microscope integrated with Nikon DS5 cooled CCD camera by 15 × objective, brightfield illumination.

### Capillary-like Tube Formation Assay

The surface of a 24-well plate was coated with Matrigel® basement membrane (50 µl/well; 10 mg/ml) on ice and was allowed to polymerize at 37°C for 1 hour. HUVEC (3×10^4^ cells) were seeded onto the coated plate in the presence of fresh conditioned media from either U-87 MG or Clone #1, complete EGM-2 medium or 10% FBS supplemented DMEM. Following 22 hours incubation, the wells were imaged using Nikon TE2000E inverted microscope integrated with Nikon DS5 cooled CCD camera by 6 × objective. The wells were analyzed using NIH ImageJ software. Total tube area was normalized to percent of tube area, with 100% representing capillary-like tube formation at the presence of DMEM supplemented with10% FBS.

### HUVEC Migration Assay

Cell migration assay was performed using transwells chambers with pore size of 8 µm (Costar Corp., Corning, NY, USA). HUVEC (2×10^5^ cells) were added to the upper chamber of 10 µg/ml fibronectin-coated 24-well transwells chambers. Following 2 hours of incubation, conditioned media from either U-87 MG or Clone #1, serum-free media (negative control) or 10% FBS-containing media (positive control) were added to the lower chamber. Cells were allowed to migrate to the lower chamber for an additional 4 hours, followed by fixation using methanol and staining (Hema 3 stain system). The stained migrated cells were imaged using a Nikon TE2000E inverted microscope integrated with Nikon DS5 cooled CCD camera by 10 × objective, brightfield illumination. Migrated cells from the captured images per membrane were counted using NIH ImageJ software.

### Matrigel Plug Angiogenesis Assay

Conditioned media from both cancer cells, U-87 MG and Clone #1, was concentrated under high vacuum Rotavapor and resuspended in 400 µl saline. Next, 80 µl of concentrated conditioned media were mixed with 600 µl growth factor-reduced liquefied Matrigel® and injected s.c. into the abdomen of 6-week-old male SCID mice. Three weeks post inoculation, vascularization within the plugs was evaluated using microbubbles contrast-enhanced ultrasound imaging.

### Animals and Tumor Cell Inoculation

Tumor cells (1×10^6^) were injected s.c. into the flank of male SCID mice aged 6–8 weeks (Charles River Laboratories, MA, USA or Harlan Laboratories, Israel). Tumor volume was calculated using the standard formula: length x width^2^ x 0.52. U-87 MG and Clone #1 human glioblastoma (5×10^4^) cells were stereotactically implanted into the striatum at the left hemisphere of SCID mice. In order to estimate tumor progression, CRI Maestro™ imaging system was used.

### Intravital Non-invasive Imaging of mCherry-labeled U-87 MG and Clone #1 Human Glioblastoma Tumor-bearing Mice

CRI Maestro™ non-invasive fluorescence imaging system was used to follow tumor progression of 6-week-old male SCID mice bearing either mCherry labeled U-87 MG or Clone #1 human glioblastoma tumors. Tumor progression was validated by caliper measurement (width^2^ × length × 0.52). Body weight and tumor size were also monitored q.o.d. (n = 3 mice/group). Mice were anesthetized using ketamine (100 mg/kg) and xylazine (12 mg/kg), treated with a depilatory cream (Veet®) and placed inside the imaging system. Multispectral image-cube were acquired through 550–800 nm spectral range in 10 nm steps using excitation (595 nm longpass) and emission (645 nm longpass) filter set. Mice autofluorescence and undesired background signals were eliminated by spectral analysis and linear unmixing algorithm.

### Intravital High-resolution Fibered Confocal Endomicroscopy of Tumor Vasculature

Endomicroscopy imaging was performed using a minimally invasive high-resolution fibered confocal microscope (CellVizio®). Mice were anesthetized and a conjugate of Dextran-FITC (70 kDa) was injected into the tail vein. Blood vessels were imaged by using a 4.2 mm-diameter optic fiber (ProFlex™ MiniO/30) and all recordings were obtained in real time.

### Microbubbles Contrast-enhanced Ultrasound Analysis of Tumor Vasculature

Ultrasound (US) imaging was performed using a Vevo2100 (VisualSonics Inc, Toronto, Canada) using the 55 MHz 708 probe. Mice were anesthetized and hair was removed over the tumor. Non-targeted microbubbles (VisualSonics Inc., Toronto, Canada) were mixed with saline and injected into the tail vein of the mice.

For US imaging of size-matched tumors: Contrast-enhanced US imaging cine loop of the tumors was acquired using the contrast scans, immediately following administration of the microbubbles. Tumor regions that are not perfused are easily discriminated from those receiving blood flow (highlighted in green) using the Vevo2100 (Visual Sonics) imaging software. Perfusion curve was calculated using the following formula:
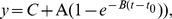



where y  =  Contrast signal (pixel intensity); A  =  Peak of curve; B  =  Slope of the curve; C  =  Contrast signal offset; t  =  Time and t_0_ =  Time offset. Blood flow within U-87 MG and Clone #1 tumors was compared using the maximum and minimum difference in microbubbles signal intensity.

For US imaging of Matrigel® plugs containing conditioned media from the cancer cells: 3D contrast-enhanced cine loop of the tumor was acquired using the 3D acquisition motor immediately after injection of the microbubbles. Following microbubbles destruction, a second 3D contrast cine loop was taken. The difference in video intensity from subtraction of the pre- and post-destruction image frames was automatically displayed by the software as a colored (green) overlay on the gray-scale images. Images were analyzed offline for 3D relative blood volume (PA) inside the tumor using the Vevo2100 imaging software.

### Immunohistochemistry

Immunohistochemistry of tumor nodules was performed using 6 mm thick formalin-fixed, paraffin-embedded tissue sections. Paraffin sections were deparaffinized, rehydrated, and stained by hematoxylin and eosin (H&E). For CD34 staining, slides were deparaffinized and pre-treated with 10 mM citrate, pH 6.0 for 50 min in a steam pressure cooker (Decloaking Chamber, BioCare Medical, Walnut Creek, CA, USA). All further steps were performed at RT in a hydrated chamber. Slides were covered with Peroxidase Block (Merck, Germany) for 5 min to quench endogenous peroxidase activity, followed by blockage of nonspecific binding sites. Blocking was performed by incubation with 10% of rabbit serum in 50 mM Tris-HCl, pH 7.4, for 30 min or 10% horse serum, 1% BSA, 0.1% triton-X and 0.05% tween-20 in PBS, pH 7.2, for 30 min. Primary rat anti-murine CD34 antibody (MEC 14.7 1:50 dilution; Abcam, Cambridge, MA, USA) and mouse anti TSP-1 antibody (1∶15 dilution; Abcam, Cambridge, MA, USA) were applied in 1% rabbit and goat serum respectively in Tris-HCl, pH 7.4 at RT for 1 hour. Slides were washed in 50 mM Tris-HCl, pH 7.4 and rabbit anti-rat antibody (1∶750 dilution; Vector Laboratories, CA, USA) was applied for 30 min, followed by anti-rabbit horseradish peroxidase-conjugate antibody (ABC detection kit, Vector Laboratories, CA, USA). Following further washing, immunoperoxidase staining was developed using ImmPACT™ DAB diluent kit (Vector Laboratories, CA, USA) per the manufacturer’s instructions and counterstained with methyl green. Microvessel density (MVD) was calculated as previously described [34]. Biotin anti-human PCNA antibody (Biolegend, San Diego, CA, USA); HRP streptavidin (Biocare medical, Concord, CA, USA). For fluorescent CD34 staining, Cy3 goat anti-rat antibody (1∶100 dilution; Invitrogen) was applied for 2 hours, followed by anti-fade mounting solution containing DAPI (Origolab, Israel).

### Statistical Methods

Data were expressed as mean ± s.d. for *in vitro* assays or ± s.e.m. for *in vivo*. Statistical significance was determined using an unpaired *t-*test. All statistical tests were two-sided. All *in vitro* experiments were performed in triplicates and repeated at least three times.

## Supporting Information

Figure S1 **Growth kinetics of tumors generated by U-87 MG parental cell line and by Clone #1.** Each line represents one tumor. Red lines indicate tumors generated from U-87 MG cell line. Blue lines indicate tumors generated from Clone #1.
(TIF)

Figure S2 **CD34 staining of size-matched (∼2 mm^3^) U-87 MG and Clone #1 tumor-sections, divided into components.**
(TIF)
