# Supplementary figures and images for: Prospective Identification of Glioblastoma Cells Generating Dormant Tumors
Source: PLoS One. 2012 Sep 6;7(9):e44395. doi: 10.1371/journal.pone.0044395 (PMC3435314; doi:10.1371/journal.pone.0044395)

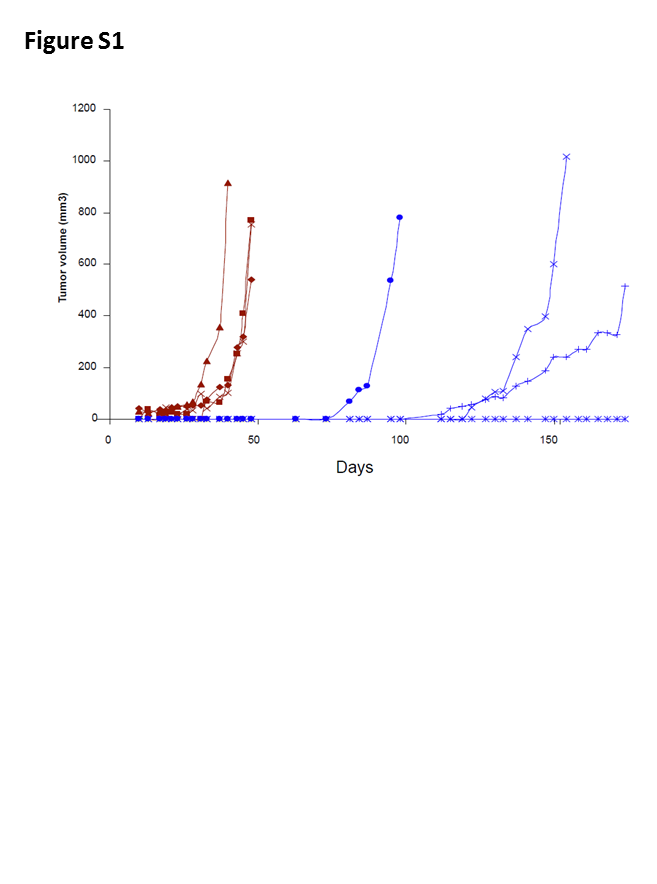

Supplement: Figure S1 — Growth kinetics of tumors generated by U-87 MG parental cell line and by Clone #1. Each line represents one tumor. Red lines indicate tumors generated from U-87 MG cell line. Blue lines indicate tumors generated from Clone #1. (TIF) [file pone.0044395.s001.tif]

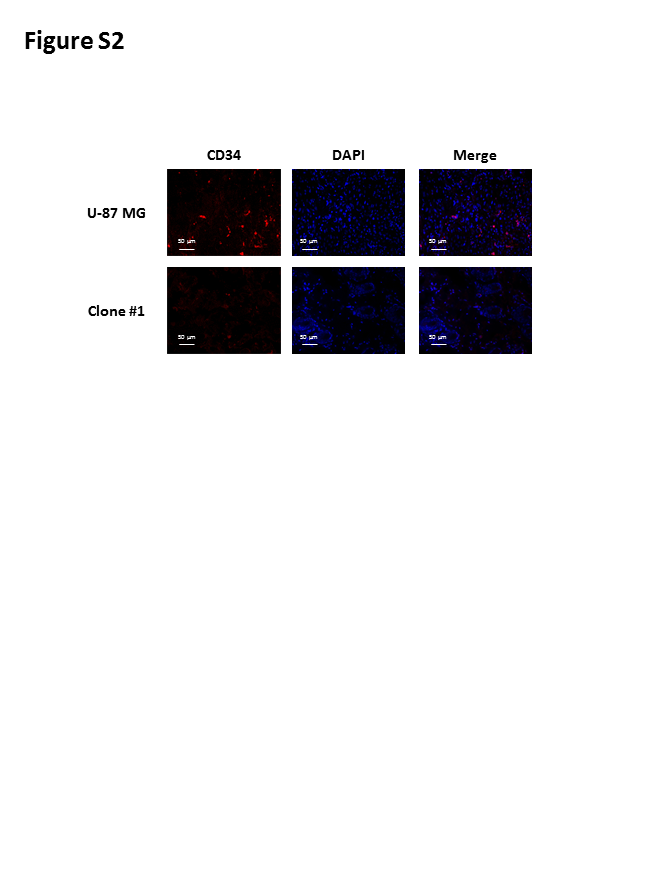

Supplement: Figure S2 — CD34 staining of size-matched (∼2 mm3) U-87 MG and Clone #1 tumor-sections, divided into components. (TIF) [file pone.0044395.s002.tif]
